# Bis[2-(benzimidazol-2-ylsulfan­yl)acetato]bis­(2,2′-bipyridine)cadmium(II)

**DOI:** 10.1107/S1600536809039592

**Published:** 2009-10-13

**Authors:** Lin Cheng, Yan-Yan Sun, Jian-Quan Wang, Ya-Wen Zhang

**Affiliations:** a School of Chemistry and Chemical Engineering, Southeast University, Nanjing 211189, People’s Republic of China

## Abstract

In the structure of the title compound, [Cd(C_9_H_7_N_2_O_2_S)_2_(C_10_H_8_N_2_)_2_], the complex mol­ecules are located on a crystallographic twofold rotation axis and the Cd^II^ ion is octa­hedrally coordinated by two chelating 2,2′-bipyridine ligands and two O atoms from the carboxyl­ate groups of two 2-(benzimidazol-2-ylsulfan­yl)acetate ligands. The two carboxyl­ate ligands adopt a *cis* configuration with respect to each other. Within each of these ligands, the imidazole fragments are bent back in a loop towards the acetyl groups, forming intra­molecular N—H⋯O hydrogen bonds, which help to stablilize the mononuclear complex. Adjacent mol­ecules are further linked by weak C—H⋯O hydrogen bonds, resulting in a chain along the *c* axis.

## Related literature

For related structures, see: Matthews *et al.* (1998 ▶); Cheng *et al.* (2009 ▶).
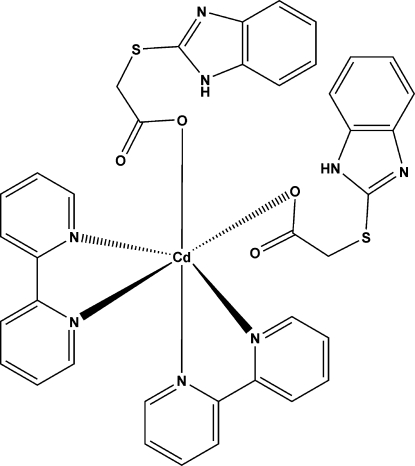


## Experimental

### 

#### Crystal data

[Cd(C_9_H_7_N_2_O_2_S)_2_(C_10_H_8_N_2_)_2_]
*M* *_r_* = 839.25
Monoclinic, 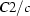

*a* = 26.733 (2) Å
*b* = 9.3043 (8) Å
*c* = 16.4220 (14) Å
β = 120.2540 (10)°
*V* = 3528.3 (5) Å^3^
*Z* = 4
Mo *K*α radiation
μ = 0.79 mm^−1^
*T* = 295 K
0.20 × 0.18 × 0.12 mm

#### Data collection

Bruker SMART CCD diffractometer
Absorption correction: multi-scan (*SADABS*; Sheldrick, 2000 ▶) *T*_min_ = 0.858, *T*_max_ = 0.911
9308 measured reflections
3460 independent reflections
2946 reflections with *I* > 2σ(*I*)
*R*_int_ = 0.028

#### Refinement

*R*[*F* ^2^ > 2σ(*F* ^2^)] = 0.030
*wR*(*F* ^2^) = 0.069
*S* = 1.05
3460 reflections
244 parameters
H atoms treated by a mixture of independent and constrained refinement
Δρ_max_ = 0.33 e Å^−3^
Δρ_min_ = −0.47 e Å^−3^

### 

Data collection: *SMART* (Bruker, 2000 ▶); cell refinement: *SAINT* (Bruker, 2000 ▶); data reduction: *SAINT*; program(s) used to solve structure: *SHELXTL* (Sheldrick, 2008 ▶); program(s) used to refine structure: *SHELXTL*; molecular graphics: *SHELXTL*; software used to prepare material for publication: *SHELXTL*.

## Supplementary Material

Crystal structure: contains datablocks I, global. DOI: 10.1107/S1600536809039592/zl2228sup1.cif

Structure factors: contains datablocks I. DOI: 10.1107/S1600536809039592/zl2228Isup2.hkl

Additional supplementary materials:  crystallographic information; 3D view; checkCIF report

## Figures and Tables

**Table 1:** Hydrogen-bond geometry (Å, °)

*D*—H⋯*A*	*D*—H	H⋯*A*	*D*⋯*A*	*D*—H⋯*A*
N2—H2*C*⋯O2	0.80 (2)	1.96 (2)	2.708 (3)	156 (2)
C11—H11*A*⋯O1^i^	0.93	2.29	3.179 (3)	161

Symmetry code: (i) 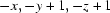.

**Fig. 1.**
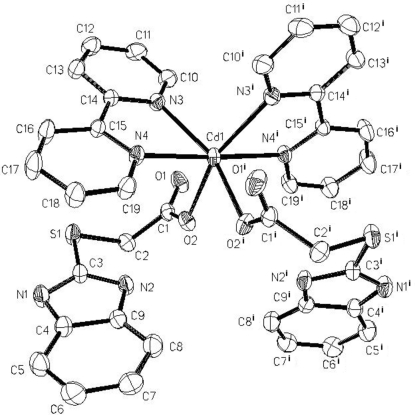
Structure of the title compound with 30% thermal ellipsoids. Symmetry code: i: -x, y, 1/2-z. Hydrogen atoms are omitted for clarity.

**Fig. 2.**
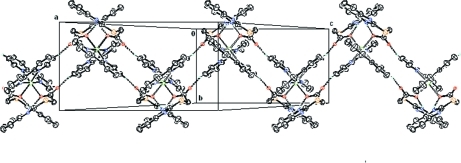
The one-dimensional hydrogen bonding chain of the title compound.

## supplementary crystallographic information

### Comment

Recently, the photophysical properties of coordination compounds of d_10_ monovalent ions of the coinage metals have attracted considerable attention. Meanwhile, benzimidazole compounds and thioether carboxylates have been widely used to construct many interesting coordination compounds. However, such compounds formed by bifunctional ligands with both benzimidazole and thioether carboxylate groups have only been rarely reported (Cheng *et al.* 2009, Matthews *et al.* 1998). Herein, we present the synthesis and structural characterization of a new coordination compound of a d_10_ mononuclear complex Cd(Hbia)_2_(2,2'-bipy)_2_ (H*2*bia = 2-(1*H*-benzo[*d*]imidazol-2-ylthio)acetic acid; 2,2'-bipy = 2,2'-bipyridine) with the bifunctional ligand H*2*bia.

In the structure of the title compound the complex is located on a crystallographic two fold rotation axis with one Cd^II^ cation, one Hbia and one chelating 2,2'-bipy ligand in the asymmetric unit. The Cd^II^ ion displays a distorted octahedral geometry, being surrounded by two chelating 2,2'-bipy ligands with Cd—N coordinating distances of 2.342 (2) and 2.378 (2) Å and two oxygen atoms coming from the carboxylates of two Hbia ligands, respectively, with the distance involving O atoms and Cd being 2.275 (2) Å. The angles around Cd are in the range of 69.50 (8)–158.51 (8) °. Meanwhile, the two carboxylate ligands are related by a two fold rotation axis and adopt a *cis-* configuration with respect to each other. Within each of these ligands the imidazole fragments are bent back in a loop towards the acetyl groups to form intramolecular N—H···O hydrogen bonds which help to stablilize the mononuclear complex (table 1). The N···O distance between N2 of the imidazole and the coordinated O atom O2 is 2.708 (3) Å. Adjacent molecules are further linked together by C—H···O hydrogen bonding between the uncoordinated oxygen atoms and the carbon atoms of 2,2'-bipyridine (C11···O1^ii^ 3.180 (4) Å. symmetry code: ^ii^, -*x*, 1-*y*, 1-*z*), resulting in a one-dimensional hydrogen bonded chain.

### Experimental

A mixture of H*2*bia (0.0208 g, 0.1 mmol), 2,2'-bipy (0.0156 g, 0.1 mmol), Cd(NO_3_)_2_.6H_2_O (0.0345 g, 0.1 mmol) and H_2_O (8 ml) was heated in a 15-ml Teflon-lined autoclave at 363 K for 5 days, followed by slow cooling (5 K h^-1^) to room temperature. The resulting mixture was washed with water, and colorless block crystals were collected and dried in air [yield 91% (76.3 mg) based on Cd(II)].

### Refinement

The H atom bonded to the N atom was located in a difference map and was freely refined without use of restraints. All other H atoms were positioned geometrically and refined using a riding model with C—H = 0.93–0.97 Å and with *U*_iso_(H) = 1.2 *U*_eq_(C).

### Crystal data

**Table:**

[Cd(C_9_H_7_N_2_O_2_S)_2_(C_10_H_8_N_2_)_2_]	*F*(000) = 1704
*M**_r_* = 839.25	*D*_x_ = 1.580 Mg m^−^^3^
Monoclinic, *C*2/*c*	Mo *K*α radiation, λ = 0.71073 Å
Hall symbol: -C 2yc	Cell parameters from 783 reflections
*a* = 26.733 (2) Å	θ = 2.4–28.0°
*b* = 9.3043 (8) Å	µ = 0.79 mm^−^^1^
*c* = 16.4220 (14) Å	*T* = 295 K
β = 120.254 (1)°	Block, colorless
*V* = 3528.3 (5) Å^3^	0.20 × 0.18 × 0.12 mm
*Z* = 4	

### Data collection

**Table:**

Bruker SMART CCD diffractometer	3460 independent reflections
Radiation source: fine-focus sealed tube	2946 reflections with *I* > 2σ(*I*)
graphite	*R*_int_ = 0.028
φ and ω scans	θ_max_ = 26.0°, θ_min_ = 1.8°
Absorption correction: multi-scan (*SADABS*; Sheldrick, 2000)	*h* = −32→30
*T*_min_ = 0.858, *T*_max_ = 0.911	*k* = −11→11
9308 measured reflections	*l* = −20→19

### Refinement

**Table:**

Refinement on *F*^2^	Primary atom site location: structure-invariant direct methods
Least-squares matrix: full	Secondary atom site location: difference Fourier map
*R*[*F*^2^ > 2σ(*F*^2^)] = 0.030	Hydrogen site location: inferred from neighbouring sites
*wR*(*F*^2^) = 0.069	H atoms treated by a mixture of independent and constrained refinement
*S* = 1.05	*w* = 1/[σ^2^(*F*_o_^2^) + (0.0305*P*)^2^ + 1.2632*P*] where *P* = (*F*_o_^2^ + 2*F*_c_^2^)/3
3460 reflections	(Δ/σ)_max_ < 0.001
244 parameters	Δρ_max_ = 0.33 e Å^−^^3^
0 restraints	Δρ_min_ = −0.47 e Å^−^^3^

### Special details

**Table:**

Geometry. All e.s.d.'s (except the e.s.d. in the dihedral angle between two l.s. planes) are estimated using the full covariance matrix. The cell e.s.d.'s are taken into account individually in the estimation of e.s.d.'s in distances, angles and torsion angles; correlations between e.s.d.'s in cell parameters are only used when they are defined by crystal symmetry. An approximate (isotropic) treatment of cell e.s.d.'s is used for estimating e.s.d.'s involving l.s. planes.
Refinement. Refinement of *F*^2^ against ALL reflections. The weighted *R*-factor *wR* and goodness of fit *S* are based on *F*^2^, conventional *R*-factors *R* are based on *F*, with *F* set to zero for negative *F*^2^. The threshold expression of *F*^2^ > σ(*F*^2^) is used only for calculating *R*-factors(gt) *etc*. and is not relevant to the choice of reflections for refinement. *R*-factors based on *F*^2^ are statistically about twice as large as those based on *F*, and *R*- factors based on ALL data will be even larger.

### Fractional atomic coordinates and isotropic or equivalent isotropic displacement parameters (Å^2^)

**Table:**

	*x*	*y*	*z*	*U*_iso_*/*U*_eq_	
Cd1	0.0000	0.27478 (3)	0.2500	0.03543 (10)	
S1	0.15271 (3)	0.05110 (8)	0.52528 (4)	0.04872 (19)	
C1	0.03416 (10)	0.1120 (3)	0.43699 (17)	0.0373 (6)	
C2	0.08216 (10)	0.0184 (3)	0.51125 (16)	0.0415 (6)	
H2A	0.0721	−0.0817	0.4945	0.050*	
H2B	0.0843	0.0346	0.5713	0.050*	
C3	0.15201 (10)	−0.0542 (3)	0.43638 (16)	0.0388 (6)	
C4	0.17937 (11)	−0.1829 (3)	0.35876 (17)	0.0413 (6)	
C5	0.20952 (13)	−0.2670 (3)	0.3279 (2)	0.0540 (7)	
H5A	0.2493	−0.2809	0.3653	0.065*	
C6	0.17850 (13)	−0.3294 (3)	0.2399 (2)	0.0567 (7)	
H6A	0.1979	−0.3860	0.2179	0.068*	
C7	0.11932 (13)	−0.3099 (3)	0.1835 (2)	0.0561 (8)	
H7A	0.1000	−0.3523	0.1242	0.067*	
C8	0.08838 (13)	−0.2290 (3)	0.2134 (2)	0.0524 (7)	
H8A	0.0486	−0.2163	0.1759	0.063*	
C9	0.11938 (10)	−0.1676 (3)	0.30194 (16)	0.0385 (6)	
C10	0.03449 (11)	0.5176 (3)	0.4126 (2)	0.0494 (7)	
H10A	−0.0049	0.5104	0.3915	0.059*	
C11	0.06748 (12)	0.6067 (3)	0.4870 (2)	0.0539 (7)	
H11A	0.0511	0.6576	0.5166	0.065*	
C12	0.12519 (12)	0.6190 (3)	0.5169 (2)	0.0526 (7)	
H12A	0.1485	0.6799	0.5667	0.063*	
C13	0.14864 (11)	0.5405 (3)	0.47287 (18)	0.0456 (6)	
H13A	0.1878	0.5479	0.4924	0.055*	
C14	0.11277 (9)	0.4506 (2)	0.39904 (16)	0.0336 (5)	
C15	0.13512 (9)	0.3589 (3)	0.35035 (16)	0.0339 (5)	
C16	0.19346 (11)	0.3488 (3)	0.3800 (2)	0.0542 (7)	
H16A	0.2203	0.4034	0.4307	0.065*	
C17	0.21161 (12)	0.2577 (3)	0.3340 (2)	0.0634 (9)	
H17A	0.2508	0.2496	0.3537	0.076*	
C18	0.17124 (11)	0.1788 (3)	0.2589 (2)	0.0525 (7)	
H18A	0.1824	0.1166	0.2266	0.063*	
C19	0.11432 (11)	0.1940 (3)	0.23272 (18)	0.0454 (7)	
H19A	0.0869	0.1410	0.1817	0.055*	
N1	0.19912 (8)	−0.1097 (2)	0.44392 (15)	0.0478 (5)	
N2	0.10299 (9)	−0.0851 (2)	0.35415 (14)	0.0410 (5)	
N3	0.05612 (8)	0.4404 (2)	0.36884 (14)	0.0390 (5)	
N4	0.09607 (8)	0.2814 (2)	0.27686 (14)	0.0360 (5)	
O1	0.01181 (8)	0.2029 (2)	0.46217 (13)	0.0559 (5)	
O2	0.02068 (7)	0.08785 (18)	0.35135 (11)	0.0422 (4)	
H2C	0.0731 (11)	−0.046 (3)	0.3391 (17)	0.040 (7)*	

### Atomic displacement parameters (Å^2^)

**Table:**

	*U*^11^	*U*^22^	*U*^33^	*U*^12^	*U*^13^	*U*^23^
Cd1	0.02273 (14)	0.04258 (17)	0.03763 (15)	0.000	0.01272 (11)	0.000
S1	0.0323 (3)	0.0634 (5)	0.0415 (4)	0.0011 (3)	0.0120 (3)	−0.0134 (3)
C1	0.0281 (12)	0.0453 (15)	0.0395 (14)	0.0012 (11)	0.0178 (11)	0.0034 (12)
C2	0.0437 (14)	0.0473 (16)	0.0351 (13)	0.0069 (12)	0.0211 (12)	0.0061 (11)
C3	0.0293 (13)	0.0463 (15)	0.0354 (13)	0.0004 (11)	0.0123 (11)	−0.0007 (11)
C4	0.0352 (13)	0.0485 (16)	0.0412 (14)	0.0000 (11)	0.0199 (12)	0.0005 (12)
C5	0.0413 (16)	0.067 (2)	0.0592 (18)	0.0054 (13)	0.0293 (15)	−0.0053 (15)
C6	0.0614 (19)	0.0594 (19)	0.0638 (19)	0.0036 (15)	0.0424 (17)	−0.0084 (16)
C7	0.064 (2)	0.0568 (19)	0.0482 (17)	−0.0032 (15)	0.0289 (15)	−0.0110 (14)
C8	0.0430 (16)	0.0599 (18)	0.0452 (16)	0.0030 (13)	0.0154 (13)	−0.0044 (14)
C9	0.0357 (13)	0.0404 (14)	0.0376 (13)	0.0031 (11)	0.0170 (11)	0.0038 (11)
C10	0.0418 (15)	0.0446 (16)	0.0701 (18)	0.0026 (12)	0.0344 (14)	−0.0066 (14)
C11	0.0633 (19)	0.0424 (16)	0.0722 (19)	0.0020 (14)	0.0462 (17)	−0.0104 (15)
C12	0.0540 (17)	0.0482 (17)	0.0573 (17)	−0.0067 (13)	0.0293 (15)	−0.0161 (14)
C13	0.0363 (14)	0.0483 (16)	0.0512 (15)	−0.0059 (12)	0.0213 (12)	−0.0138 (13)
C14	0.0288 (12)	0.0340 (13)	0.0379 (13)	0.0002 (10)	0.0167 (11)	0.0002 (10)
C15	0.0275 (12)	0.0379 (14)	0.0362 (13)	−0.0033 (10)	0.0160 (10)	−0.0032 (11)
C16	0.0296 (13)	0.070 (2)	0.0594 (17)	−0.0091 (13)	0.0196 (13)	−0.0281 (15)
C17	0.0293 (14)	0.088 (2)	0.073 (2)	−0.0054 (14)	0.0259 (15)	−0.0318 (18)
C18	0.0393 (15)	0.0666 (19)	0.0589 (17)	−0.0006 (13)	0.0302 (14)	−0.0177 (15)
C19	0.0373 (14)	0.0567 (18)	0.0443 (15)	−0.0087 (12)	0.0220 (12)	−0.0163 (13)
N1	0.0295 (11)	0.0618 (15)	0.0459 (13)	0.0042 (10)	0.0144 (10)	−0.0054 (11)
N2	0.0281 (11)	0.0496 (14)	0.0393 (12)	0.0059 (10)	0.0126 (10)	−0.0043 (10)
N3	0.0297 (11)	0.0404 (12)	0.0491 (12)	0.0008 (9)	0.0215 (10)	−0.0055 (10)
N4	0.0267 (10)	0.0443 (12)	0.0366 (11)	−0.0027 (9)	0.0156 (9)	−0.0065 (9)
O1	0.0470 (11)	0.0704 (14)	0.0491 (11)	0.0212 (10)	0.0234 (9)	−0.0027 (10)
O2	0.0393 (9)	0.0521 (11)	0.0332 (9)	0.0121 (8)	0.0168 (8)	0.0054 (8)

### Geometric parameters (Å, °)

**Table:**

Cd1—O2^i^	2.2746 (16)	C8—H8A	0.9300
Cd1—O2	2.2746 (16)	C9—N2	1.376 (3)
Cd1—N3	2.342 (2)	C10—N3	1.336 (3)
Cd1—N3^i^	2.3417 (19)	C10—C11	1.369 (4)
Cd1—N4^i^	2.3775 (19)	C10—H10A	0.9300
Cd1—N4	2.3775 (19)	C11—C12	1.368 (4)
S1—C3	1.750 (2)	C11—H11A	0.9300
S1—C2	1.807 (2)	C12—C13	1.381 (3)
C1—O1	1.221 (3)	C12—H12A	0.9300
C1—O2	1.284 (3)	C13—C14	1.385 (3)
C1—C2	1.521 (3)	C13—H13A	0.9300
C2—H2A	0.9700	C14—N3	1.338 (3)
C2—H2B	0.9700	C14—C15	1.485 (3)
C3—N1	1.308 (3)	C15—N4	1.340 (3)
C3—N2	1.357 (3)	C15—C16	1.383 (3)
C4—C5	1.389 (4)	C16—C17	1.375 (4)
C4—N1	1.398 (3)	C16—H16A	0.9300
C4—C9	1.398 (3)	C17—C18	1.373 (4)
C5—C6	1.381 (4)	C17—H17A	0.9300
C5—H5A	0.9300	C18—C19	1.364 (3)
C6—C7	1.385 (4)	C18—H18A	0.9300
C6—H6A	0.9300	C19—N4	1.335 (3)
C7—C8	1.378 (4)	C19—H19A	0.9300
C7—H7A	0.9300	N2—H2C	0.80 (2)
C8—C9	1.383 (4)		
			
O2^i^—Cd1—O2	80.25 (8)	N2—C9—C4	105.0 (2)
O2^i^—Cd1—N3	158.50 (6)	C8—C9—C4	122.5 (2)
O2—Cd1—N3	94.35 (7)	N3—C10—C11	123.2 (2)
O2^i^—Cd1—N3^i^	94.35 (7)	N3—C10—H10A	118.4
O2—Cd1—N3^i^	158.50 (6)	C11—C10—H10A	118.4
N3—Cd1—N3^i^	97.71 (10)	C12—C11—C10	118.3 (2)
O2^i^—Cd1—N4^i^	92.44 (6)	C12—C11—H11A	120.9
O2—Cd1—N4^i^	89.84 (6)	C10—C11—H11A	120.9
N3—Cd1—N4^i^	108.43 (7)	C11—C12—C13	119.7 (3)
N3^i^—Cd1—N4^i^	69.49 (6)	C11—C12—H12A	120.1
O2^i^—Cd1—N4	89.84 (6)	C13—C12—H12A	120.1
O2—Cd1—N4	92.44 (6)	C12—C13—C14	118.8 (2)
N3—Cd1—N4	69.49 (6)	C12—C13—H13A	120.6
N3^i^—Cd1—N4	108.43 (7)	C14—C13—H13A	120.6
N4^i^—Cd1—N4	177.01 (10)	N3—C14—C13	121.5 (2)
C3—S1—C2	103.15 (12)	N3—C14—C15	116.5 (2)
O1—C1—O2	125.2 (2)	C13—C14—C15	122.0 (2)
O1—C1—C2	118.9 (2)	N4—C15—C16	120.6 (2)
O2—C1—C2	115.8 (2)	N4—C15—C14	116.84 (19)
C1—C2—S1	114.25 (17)	C16—C15—C14	122.5 (2)
C1—C2—H2A	108.7	C17—C16—C15	119.7 (2)
S1—C2—H2A	108.7	C17—C16—H16A	120.1
C1—C2—H2B	108.7	C15—C16—H16A	120.1
S1—C2—H2B	108.7	C18—C17—C16	119.2 (3)
H2A—C2—H2B	107.6	C18—C17—H17A	120.4
N1—C3—N2	114.3 (2)	C16—C17—H17A	120.4
N1—C3—S1	122.47 (18)	C19—C18—C17	118.3 (2)
N2—C3—S1	123.23 (18)	C19—C18—H18A	120.8
C5—C4—N1	130.0 (2)	C17—C18—H18A	120.8
C5—C4—C9	119.7 (2)	N4—C19—C18	123.2 (2)
N1—C4—C9	110.2 (2)	N4—C19—H19A	118.4
C6—C5—C4	117.8 (3)	C18—C19—H19A	118.4
C6—C5—H5A	121.1	C3—N1—C4	103.73 (19)
C4—C5—H5A	121.1	C3—N2—C9	106.7 (2)
C5—C6—C7	121.7 (3)	C3—N2—H2C	121.9 (18)
C5—C6—H6A	119.1	C9—N2—H2C	130.3 (18)
C7—C6—H6A	119.1	C10—N3—C14	118.6 (2)
C8—C7—C6	121.5 (3)	C10—N3—Cd1	122.20 (16)
C8—C7—H7A	119.3	C14—N3—Cd1	119.05 (15)
C6—C7—H7A	119.3	C19—N4—C15	119.0 (2)
C7—C8—C9	116.8 (3)	C19—N4—Cd1	122.67 (16)
C7—C8—H8A	121.6	C15—N4—Cd1	117.36 (14)
C9—C8—H8A	121.6	C1—O2—Cd1	119.90 (16)
N2—C9—C8	132.4 (2)		

Symmetry codes: (i) −*x*, *y*, −*z*+1/2.

### Hydrogen-bond geometry (Å, °)

**Table:**

*D*—H···*A*	*D*—H	H···*A*	*D*···*A*	*D*—H···*A*
N2—H2C···O2	0.80 (2)	1.96 (2)	2.708 (3)	156 (2)
C11—H11A···O1^ii^	0.93	2.29	3.179 (3)	161

Symmetry codes: (ii) −*x*, −*y*+1, −*z*+1.
